# Sodium-Glucose Cotransporter-2 Inhibitor and Glucagon-Like Peptide-1 Receptor Agonist Combination Therapy in Type 2 Diabetes: Protocol for a Kidney End Points Real-world Study (COMBi-KID Study)

**DOI:** 10.2196/34206

**Published:** 2022-07-19

**Authors:** Michael Feher, William Hinton, Anna Forbes, Neil Munro, Mark Joy, David Wheeler, Simon de Lusignan

**Affiliations:** 1 Clinical Informatics and Health Outcomes Research Group Nuffield Department of Primary Care Health Sciences University of Oxford Oxford United Kingdom; 2 Clinical Informatics University of Surrey Guildford United Kingdom; 3 Department of Renal Medicine University College London London United Kingdom

**Keywords:** type 2 diabetes, sodium-glucose cotransporter-2 inhibitor, glucagon-like peptide-1 receptor agonist, renal, kidney, electronic health records

## Abstract

**Background:**

Sodium-glucose cotransporter-2 inhibitors (SGLT2is) and glucagon-like peptide-1 receptor agonists (GLP-1RAs) are both considered to be part of standard care in the management of glycemia in type 2 diabetes. Recent trial evidence has indicated benefits on primary kidney end points for individual drugs within each medication class. Despite the potential benefits of combining SGLT2is and GLP-1RAs for glycemia management, according to national and international guideline recommendations, there is currently limited data on kidney end points for this drug combination.

**Objective:**

The aims of the study are to assess the real-world effects of combination SGLT2i and GLP-1RA therapies for diabetes management on kidney end points, glycemic control, and weight in people with type 2 diabetes who are being treated with renin-angiotensin system blockade medication.

**Methods:**

This retrospective cohort study will use the electronic health records of people with type 2 diabetes that are registered with general practices covering over 15 million people in England and Wales and are included in the Oxford-Royal College of General Practitioners Research and Surveillance Centre network. A propensity score–matched cohort of prevalent new users of SGLT2is and GLP-1RAs and those who have been prescribed SGLT2is and GLP-1RAs in combination will be identified. They will be matched based on drug histories, comorbidities, and demographics. A repeated-measures, multilevel, linear regression analysis will be performed to compare the mean change (from baseline) in estimated glomerular filtration rate at 12 and 24 months between those who switched to combined therapy and those continuing monotherapy with an SGLT2i or GLP-1RA. The secondary end points will be albuminuria, serum creatinine level, glycated hemoglobin level, and BMI. These will also be assessed for change at the 12- and 24-month follow-ups.

**Results:**

The study is due to commence in March 2022 and is expected to be complete by September 2022.

**Conclusions:**

Our study will be the first to assess the impact of combination SGLT2i and GLP-1RA therapy in type 2 diabetes on primary kidney end points from a real-world perspective.

**International Registered Report Identifier (IRRID):**

PRR1-10.2196/34206

## Introduction

### Background

Diabetes continues to be the leading cause of chronic kidney disease (CKD) and end-stage kidney disease (ESKD) requiring renal replacement therapy. Overall, about 40% of people with type 2 diabetes have evidence of diabetic kidney disease (DKD) [1-4]. For the last 2 decades, renin-angiotensin system (RAS) blockade medications (either angiotensin-converting enzyme inhibitors or angiotensin receptor blockers) have been considered—in addition to maximal glucose and blood pressure control—to be part of standard therapy for preventing or delaying the progression of DKD [1-4]. Yet, the incidence and prevalence of residual kidney disease, including CKD and ESKD, remain high and are associated with the high costs of care and dialysis [1-4]. Recent evidence from intervention outcome trials with sodium-glucose cotransporter-2 inhibitors (SGLT2is) and glucagon-like peptide-1 receptor agonists (GLP-1RAs) have indicated that these drugs individually have clear kidney benefits [1,5-15].

In cardiovascular outcome trials with SGLT2is and GLP-1Ras, which were used in the management of type 2 diabetes [1,5-15], several drugs from both classes showed benefits beyond glucose control, such as reducing the risk of heart failure and decreasing cardiovascular morbidity and mortality [1,5-15]. Moreover, it has been demonstrated that SGLT2is reduce the risk of new and worsening DKD [1,5-11]. There are also emerging data on kidney end points in GLP-1RA therapy trials, which have shown benefits on albumin excretion and reductions in estimated glomerular filtration rate (eGFR) decline [1,12-15].

Despite the potential benefits of combining SGLT2is and GLP-1RAs [16], according to guideline recommendations for the management of glycemia [17,18], there is currently limited data on kidney end points for this combination. This protocol describes our proposed study, which will explore whether combination SGLT2i and GLP-1RA therapy has additional kidney benefits when compared to treatment with either medication separately in real-world clinical practice.

### Aims and Objectives

The aim of our study is to explore the real-world effectiveness of combining SGLT2i and GLP-1RA therapies in people with type 2 diabetes who are being treated with RAS blockade medication.

The primary objective is to assess the kidney effects (eGFR change) of combining SGLT2i and GLP-1RA therapies in the management of glycemia among people with type 2 diabetes who are being treated with RAS blockade medication. The secondary objectives are to assess the effects on albuminuria, glycemic control, and weight resulting from combining SGLT2i and GLP-1RA therapies among people with type 2 diabetes who are being treated with RAS blockade medication.

## Methods

### Study Design

Our study will be a retrospective observational cohort study that uses computerized medical records (CMRs) from general practices in England and Wales that contribute to the Oxford-Royal College of General Practitioners (RCGP) Research and Surveillance Centre (RSC) network.

### Recruitment

The Oxford-RCGP RSC network is an established national database, is representative of patients attending primary care in England and Wales [19], and comprises CMR data from over 1800 urban and nonurban general practices. The Oxford-RCGP RSC database contains over 15 million patients, including over 900,000 people with diabetes, and includes demographic, coded diagnostic, laboratory test, and prescription data for men and women with diabetes in our study age band [19,20]. The size of the network has expanded considerably during the COVID-19 pandemic, and Oxford and the RCGP RSC are evolving into a major digital hub—the Oxford RCGP Clinical Informatics Digital Hub [21,22].

The primary data will be obtained from CMRs that use clinical codes. Until recently, the main terminology coding system that was used was the Read system (Read version 2 and Clinical Terms version 3). This has been replaced with the Systematized Nomenclature of Medicine Clinical Terms system, which uses clinical codes for diagnoses, prescriptions, investigations, and processes of care.

Data completeness in the RCGP RSC database is high for type 2 diabetes data due to the pay-for-performance incentive program for improving the coding of chronic diseases [20,23], the Quality and Outcomes Framework, and a dedicated team of practice liaison officers who are working closely with general practices and are able to provide feedback on coding. In addition to having high-quality data, the database also keeps its data up to date by performing updates every 3 to 10 days.

The Oxford-RCGP RSC network, as a national research platform [19-21,24], is also unique because there is no research license fee, and the network provides direct support for design-specific topics.

The study population will be adults with a diagnosis of type 2 diabetes, and we will identify this cohort by using a previously described 2-step ontological approach [20,24].

### Inclusion Criteria

The inclusion criteria are as follows:

- Adults >40 to <80 years of age
- Diagnosed type 2 diabetes (>3 months)
- eGFR of >45 mL/min/1.73 m^2^ (this is the lower eGFR limit on the product labels of SGLT2is for glycemia management) [25]
- Current therapy (>3 months) to include an RAS blocker (either an angiotensin-converting enzyme inhibitor or an angiotensin receptor blocker)
- Current diabetes therapy (>3 months) to include either an SGLT2i (dapagliflozin, empagliflozin, ertugliflozin, or canagliflozin) or a GLP-1RA (exenatide, lixisenatide, liraglutide, dulaglutide, or semaglutide)
- A minimum of 1 baseline creatinine measurement within 24 months of study entry and 1 creatinine measurement during study follow-up (24 months)

### Exclusion Criteria

The exclusion criterion is diagnosed type 1 diabetes.

### Exposures

The primary exposures of interest in our study will be a current prescription for (1) an SGLT2i (excluding GLP-1RAs), (2) a GLP-1RA (excluding SGLT2is), or (3) a combination of an SGLT2i and GLP-1RA.

We will conduct a retrospective cohort study from the time of the coprescription of an SGLT2i and GLP-1RA combination, and paired groups for diabetes therapy will include patients taking SGLT2is (but not GLP-1RAs) and patients taking GLP-1RAs (but not SGLT2is). The follow-up time for each group will be 12 months (time window: +3 or −3 months) and 24 months (time window: +3 or −3 months) following the initiation of SGLT2i and GLP-1RA combination therapy, and comparator groups will include patients undergoing either SGLT2i monotherapy or GLP-1RA monotherapy.

The clinical and biochemistry variables that will be assessed include weight (kg), hemoglobin A_1c_ (HbA_1c_; mmol/mol and %), the urine albumin-creatinine ratio (UACR), and the eGFR (mL/min/1.73 m^2^), which are recorded as part of routine clinical care. Creatinine measurements will be used to calculate eGFRs via the CKD Epidemiology Collaboration equation [26,27].

### Variables Adjusted in Matching

The variables that will be adjusted during the matching process include age, sex, ethnicity, weight, blood pressure, cardiovascular disease, medication persistence (*nonpersistence* is defined as a gap of ≥90 days in prescription [24]), diuretic or nonsteroidal anti-inflammatory drug use (>3 months), the duration of diabetes, HbA_1c_ level, eGFR, and the UACR.

### Outcomes of Interest

#### Primary End Point

The primary end point will be eGFR (mL/min/1.73 m^2^) change over time.

#### Secondary End Points

The secondary end points will include the following:

- UACR
- eGFR (mL/min/1.73 m^2^) change over time for cohorts with a baseline eGFR of (1) ≥60 mL/min/1.73 m^2^ and (2) 45 to 59 mL/min/1.73 m^2^
- Serum creatinine level
- HbA_1c_ level
- Weight (BMI)

### Statistical Analyses

The prevalent new-user design will proceed as follows. A cohort of all individuals who have been prescribed SGLT2is and/or GLP-1RAs will form a base cohort. As individuals can switch to combined SGLT2i and GLP-1RA therapy, we will identify an exposure set consisting of individuals who were exposed to either SGLT2is only or GLP-1RAs only prior to being prescribed combination therapy. The exposure set of individuals will constitute potential matches—participants who share the same drug histories, comorbidities, and demographics as those of given “switchers” (participants who switched to combined therapy). The prescription-based exposure sets will therefore provide equivalent points in the disease course with regard to comparator drug history and equivalent points at which confounder patient characteristics can be measured. A considerable computational challenge will arise when using estimated hazards as balancing scores in the matching process, given that there are approximately 3000 users of combined therapy in the Oxford-RCGP RSC data set. This will give rise to 3000 exposure sets with around 25,000 individuals. We will take (for sensitivity analyses) 10, 20, and 100 random prescription histories from each exposure set to estimate time-dependent propensity scores for switching therapies via conditional logistic regression (histories will be matched for each exposure set), whereby relative odds will be used to accurately estimate the corresponding relative hazards. The estimated propensity odds scores of the index “switchers” will be used to identify matched individuals (ie, participants with the closest matching variable values) from all members of the exposure set (not just the sampled members).

Summary statistics will be reported by using counts and percentages for categorical data, while means (with SDs) will be used to describe continuous data. We will report baseline demographics and comorbidities (using a chi-square test of independence for categorical variables and a Kruskal-Wallis test of difference for continuous variables) in the base cohort and in the matched cohort and adjudge whether good matching in the latter cohort has been achieved based on a standardized mean difference of <0.1 between groups. We will calculate and report mean changes (with SDs) in eGFR, albuminuria, serum creatinine level, HbA_1c_ level, and BMI between groups in the base cohort.

We will multiply impute (using the chained equations method) any missing data. A sensitivity analysis will be conducted on complete cases only. The primary analysis will include all participants. We will use a repeated-measures, multilevel, linear regression (with measurement occasions nested within individuals) to compare mean changes (from baseline) in eGFR at 12 and 24 months between those who switched to combined therapy and those on a single drug. A base model (containing only a cohort indicator) and a fully adjusted model (containing a cohort indicator together with all study variables) will be constructed and presented for inferences.

Secondary end points (albuminuria, serum creatinine level, HbA_1c_ level, and BMI) will be similarly assessed for change at follow-ups.

The baseline eGFR will be a covariate, since we are using a repeated-measure regression for analyzing changes in eGFR from baseline to 12 and 24 months. Albuminuria, as a secondary outcome, will also be a covariate in the repeated-measure regression for analyzing changes from baseline to 12 and 24 months.

### Power Calculation

We used G*Power to perform a power calculation for a repeated-measures ANOVA, basing our calculation on the results of a trial [13] where changes in eGFRs over 52 weeks were compared between drug groups. An absolute difference in eGFR reduction was measured at 2.7 mL/min/1.73 m^2^ between drug groups. Given that an SE of 0.7 was reported for eGFR values in both groups and assuming a sample size of 384, we estimated a Cohen effect size (*f*_z_) of approximately 0.19 (a moderate effect size) with 90% power at an α level of .05 for detecting a between-group effect of 0.10 (assuming a correlation of 0.5 between repeated measures). As such, we require a (total) sample size of 1032. Such a sample size is also sufficient for detecting group-by-time interaction effects of the same size.

### Ethics Approval

The study proposal was approved by the Medical Sciences Interdivisional Research Ethics Committee, University Oxford, in August 2021 (approval number: R76885/RE001).

## Results

The study is due to commence in March 2022 and is expected to be complete by September 2022.

## Discussion

### Study Implications

Our study will evaluate if combining 2 glucose-lowering drugs with established kidney benefits in randomized clinical trials and with differing mechanisms of action will have additive effects on kidney end points in real-world clinical practice.

Diabetes continues to be a leading cause of CKD and ESKD. Following the studies that were conducted 2 decades ago on the renal benefits of RAS blockers, these drugs are now considered part of standard therapy for preventing or delaying the progression of DKD [1]. The recent evidence for SGLT2i and GLP-1RA therapy with regard to their individual positive benefits on kidney end points has offered further individual drug options with renoprotective mechanisms in type 2 diabetes [5-15].

The mechanisms by which both drug classes influence kidney end points, such as reducing the risk and progression of albuminuria and slowing eGFR decline in type 2 diabetes, remain to be fully elucidated [28-30] Both drug groups favorably affect major risk factors for developing CKD by improving hyperglycemia, blood pressure, and weight loss. Further, trials reporting the effects of SGLT2is and GLP-1RAs on the progression of renal parameters have shown that these benefits occur independently of other clinical factors. Recent evidence indicates that GLP-1RAs have direct antiatherosclerotic influences on antioxidant, anti-inflammatory, and antifibrotic effects in diabetic kidneys [28]. By contrast, SGLT2is exert a hemodynamic effect, as well as specific, intrarenal, hemodynamic changes, that may protect glomeruli from high-pressure damage [29]. There is emerging physiological evidence for a combination effect [30].

### Strengths and Limitations of the Study

The strengths of the study is that the Oxford-RCGP RSC database is derived from a large sample size with wide national coverage across primary care in England. The other benefits of the database include high data quality, with data dating back to 2004, which makes the database an ideal resource for the longitudinal follow-up of patient populations. The Oxford-RCGP RSC network also comprises a broadly representative population in terms of age, sex, and ethnicity when compared to England and Wales census data.

A limitation of the study is confounding. The prevalent new-user study design and time-conditional propensity score matching are used to address this, but there may be residual confounding due to unmeasured variables.

The process for considering an individual who is exposed to the medication of interest from the date of the first prescription (cohort entry) until the date of the last prescription plus 3 months is a potential limitation of the study. First, there is an assumption that the participants are taking the medication as prescribed, and second, the participants may have temporarily discontinued the medication during the presumed exposure period. Finally, the 3-month grace period relates to the longest prescription that a general practitioner can issue to a patient but is likely to be an overestimate in most cases.

Time-related biases are a potential limitation of the study. Time-lag bias may arise as a consequence of SGLT2i, GLP-1RA, and combination treatment not being initiated at the same time on the diabetes pathway. Consequently, the exposure groups may be at different stages of diabetes. We have attempted to address this by including the duration of diabetes and diabetic retinopathy—a microvascular complication of diabetes—in the propensity score.

### Conclusions

There is emerging evidence from observational studies on the generalizability of cardiovascular outcome trials involving either SGLT2i monotherapy or GLP-1RA monotherapy to real-world clinical practice [31,32]. Our study will be one of the first studies to assess the effects of SGLT2i and GLP-1RA combination therapy in the management of type 2 diabetes and its effects on kidney end points from a real-world clinical perspective.

## Abbreviations

CKD: chronic kidney disease
CMR: computerized medical record
DKD: diabetic kidney disease
eGFR: estimated glomerular filtration rate
ESKD: end-stage kidney disease
GLP-1RA: glucagon-like peptide-1 receptor agonist
HbA_1c_: hemoglobin A_1c_
RAS: renin-angiotensin system
RCGP: Royal College of General Practitioners
RSC: Research and Surveillance Centre
SGLT2i: sodium-glucose cotransporter-2 inhibitor
UACR: urine albumin-creatinine ratio
